# A Large Language Model-Based Simulation Protocol Using ChatGPT for Training Novice Critical Care Nurses in Family Crisis Communication and De-escalation

**DOI:** 10.7759/cureus.114402

**Published:** 2026-08-12

**Authors:** Dimitrios Alefragkis, Dimitra Aloizou, Vasiliki Chougia

**Affiliations:** 1 Second Department of Critical Care, Attikon University Hospital, National and Kapodistrian University of Athens, Athens, GRC; 2 Department of Nursing, School of Health Sciences, National and Kapodistrian University of Athens, Athens, GRC; 3 Intensive Care Unit, General Hospital of Nikaia-Piraeus "Agios Panteleimon", Nikaia, GRC

**Keywords:** artificial intelligence, chatgpt, communication skills, critical care nursing, family-centered care, large language models, nursing education, simulation in medical education, verbal de-escalation, virtual patient simulations

## Abstract

High-stress conversations with family members in distress are a common part of the intensive care unit (ICU) nursing environment. Novice critical care nurses may have limited opportunities to practice these interactions before encountering them in clinical care. Traditional simulation methods can support communication training but often require faculty time, standardized patients, programming, and specialized resources. This technical report presents a structured simulation protocol using ChatGPT (OpenAI OpCo, LLC, San Francisco, CA, USA) to support formative practice in family crisis communication and verbal de-escalation. The protocol uses a fictional ICU scenario in which the trainee communicates with an AI-generated persona. This persona represents a mother in distress at the ICU entrance after visiting hours. The simulated family member is asked to respond dynamically to the learner’s communication style, de-escalating when the learner uses empathy, active listening, and clear boundaries and escalating only when the learner uses defensive, dismissive, or overly technical language. At the end of the session, ChatGPT provides formative feedback in six areas of communication. The areas are empathy, active listening, clarity, boundary setting, avoidance of defensive language, and overall de-escalation. This protocol is not intended to replace supervised simulation, standardized patient encounters, clinical mentorship, or real-world communication experience. Rather, it offers a cost-effective, repeatable, and privacy-protected practice that can complement communication skills training for beginning critical care nurses.

## Introduction

The intensive care unit (ICU) is a high-acuity, fast-paced environment in which healthcare professionals are required to collaborate seamlessly, while clinical uncertainty, restricted access, and volatile shifts in patient status often trigger severe emotional distress for families. Relatives of critically ill patients require timely updates, clear reassurance, and a structured role in care decisions. This forces the multidisciplinary team to balance transparent communication and empathy with the privacy and operational safety of the unit. Current guidelines for family-centered care place great emphasis on characteristics such as communication, shared decision-making, and family support as essential elements of high-quality intensive care practice [1-3].

As frontline clinicians, ICU nurses are typically the first professionals families encounter at the bedside or upon entering the unit. Managing these highly charged interactions poses a notorious challenge for novice nurses, as effective crisis communication demands far more than delivering clinical data. It includes non-technical skills such as active listening, emotional regulation, empathy, providing social support, setting boundaries, and cultural sensitivity. Generally, nurses develop these competencies over time through active clinical exposure, targeted feedback, and reflective practice. Still, early-career nurses lack sufficient or frequent training; their responses often become overly procedural, defensive, or rigid, increasing the risk of misunderstanding, conflict, and stress for both families and staff, resulting in reduced trust and psychological strain for the family [1-5].

Rapid evolution in generative AI and large language models (LLMs) opens novel pathways for healthcare education across multiple fields. LLMs are generative AI systems that can produce human-like text responses based on user prompts and prior conversational context. Specifically, educators have utilized ChatGPT to streamline clinical simulation design and drive nursing initiatives, including intensive care nursing education and practice [6-9]. Emerging data show that LLM-powered simulated patients and virtual hospital environments provide flexible opportunities for communication training and automated feedback in healthcare professional education [10, 11]. Despite these technological advantages, nursing curricula still lack structured, reproducible protocols capable of translating the AI capabilities into practical, standardized training scenarios [12, 13]. Unlike broader AI-assisted simulation approaches, the present protocol focuses specifically on ICU family crisis communication and verbal de-escalation by providing a complete scenario, system prompt, workflow, feedback rubric, and privacy safeguards.

To address this gap, this technical report outlines a structured ChatGPT-based (OpenAI OpCo, LLC, San Francisco, CA, USA) simulation protocol designed to help novice critical care nurses practice family crisis communication and verbal de-escalation. This takes place in a safe, low-cost, and infinitely repeatable learning environment. The framework details a standardized ICU scenario, a comprehensive system prompt for simulating a distressed relative, a step-by-step operational workflow, a formative evaluation rubric, and essential privacy safeguards responsible for AI implementation.

## Technical report

Tools

This protocol was designed for use with ChatGPT, an LLM-based conversational interface developed by OpenAI OpCo, LLC, San Francisco, CA, USA. The protocol is model-flexible and can be implemented using GPT-4 or later versions of ChatGPT, provided that the same scenario, system prompt, learner instructions, and feedback rubric are consistently applied [6,7]. It is important to note that the learner does not need to have programming skills, specialized software, institutional simulation equipment, or access to patient data.

The protocol is intended as a supplemental tool for training novice critical care nurses. It is not a replacement for faculty supervision, clinical mentoring, standardized patient encounters, or formal communication skills training that may be provided by the healthcare organization [8,9]. The purpose of this tool is to provide beginning critical care nurses with a cost-effective and repeatable opportunity to practice communication strategies and skills in a simulated encounter with an emotionally distressed family member who presents barriers to communication. The simulation is text-based and focuses on verbal communication, empathy, boundary setting, and de-escalation strategies where needed. Most importantly, no actual patient data is entered into the system at any stage, as this would raise ethical and privacy concerns [14,15].

Protocol design

The protocol was developed as a structured simulation model for a single learner, consisting of three phases, which are the preparation phase, the interaction phase, and finally the feedback phase. During the preparation phase, the learner is initially provided with the clinical context, learning objectives, and safety rules. In the interaction phase, ChatGPT takes on the role of a virtual distressed family member and responds dynamically to the learner’s written communication. During the feedback phase, the simulation is interrupted, and ChatGPT provides structured feedback based on predefined communication criteria.

The protocol is based on a persona-based prompting approach. ChatGPT is instructed to portray a specific family member with a defined emotional state, clinical context, behavioral pattern, and response rules based on the learner’s responses. The model is also instructed to provide a dynamic environment and to escalate or de-escalate its responses according to the learner's communication style [6,10]. When given to the model, empathetic, calm, and validating responses should gradually reduce hostility, while defensive, dismissive, overly technical, or threatening responses should increase resistance and emotional tension. Essentially, this design allows the learner to observe the effects of different communication strategies and skills in a controlled and safe learning environment [4,5].

The protocol's learning objectives were formulated according to Bloom's revised taxonomy [16]. The protocol aims to help novice critical care nurses identify signs of emotional distress in patients' family members by interpreting verbal and nonverbal communication; use active listening techniques and emotion-validation statements when communicating with patients' relatives; set clear and respectful professional boundaries in their interactions with family members; use non-defensive and non-confrontational language, avoiding conflicts in high-stress situations; and evaluate their personal communication performance, utilizing structured feedback to identify areas for improvement.

The learning objectives are summarized in Table 1 below.

**Table 1:** Learning objectives of the ChatGPT-based simulation protocol. The table maps the core training domains to Bloom's revised taxonomy [16] action verbs, cognitive process levels, and specific communication behaviors expected from the novice nurse during the simulated encounter.

Learning objective	Bloom’s action verb	Cognitive process level	Expected learner behavior during the simulation
Recognizing emotional distress	Identify/Interpret	Understanding	Recognizes verbal cues of anxiety, anger, fear, and loss of control in the simulated family member.
Demonstrating empathy and active listening	Use/Implement	Applying	Responds with validating statements, acknowledges emotions, and avoids interrupting or dismissing the family member.
Establishing professional boundaries	Set/Establish	Applying	Explains ICU rules and visiting limitations clearly, respectfully, and without escalating the interaction.
Avoiding defensive communication	Use/Apply	Applying	Avoids authoritarian, dismissive, overly technical, or confrontational language during the conversation.
Reflecting on communication performance	Evaluate/Assess	Evaluating	Reviews the structured feedback and identifies strengths, weaknesses, and areas for improvement.

This protocol is a protected practice space focusing exclusively on communication skills. It does not assess medical knowledge and does not replace face-to-face interaction with relatives. Its purpose is to support novice critical care nurses.

Simulation scenario

The simulation scenario represents a high-stress communication encounter at the ICU entrance. In this scenario, a distressed family member requests immediate access to a critically ill patient after visiting hours have ended. The learner must respond to the relative’s emotional distress while maintaining professional boundaries, respecting unit policy, and preventing further escalation. The simulated family member is a 50-year-old mother whose 22-year-old son was admitted to the ICU four hours earlier following a severe motor vehicle accident. The afternoon visiting period has ended, and she has been asked to leave the ICU entrance. She refuses to leave, raises her voice, and demands to see her son. She expresses various emotions, including fear, anger, and distrust toward the healthcare team. The learner assumes the role of a novice critical care nurse and is expected to respond using calm, empathic, and structured communication.

The scenario was intentionally designed without real patient information, specific institutional details, or complex clinical decision-making. Its main purpose is not to test medical knowledge or provide clinical recommendations, but to create a repeatable communication exercise. The emotional intensity of the simulated family member changes according to the learner’s response style. Empathic and validating responses are expected to reduce hostility, while defensive, dismissive, overly technical, or confrontational responses are expected to increase emotional resistance.

System prompt

The simulation is initialized by entering a predefined system prompt into ChatGPT. The aim of the prompt is to create a consistent simulated family-member persona, define the emotional context of the encounter, and establish clear response rules. The rules are used for escalation, de-escalation, and feedback. The prompt should be entered before the learner begins the role-play. The same prompt should be used across repeated sessions to improve consistency, unless the educator intentionally modifies the scenario difficulty. The complete system prompt is presented in Table 2.

**Table 2:** System prompt for the simulated distressed family member. The prompt outlines the specific behavioral rules, emotional escalation scales, and safety boundaries required to initialize the ChatGPT-based simulation.

Prompt component	Instruction
Role	Act as a 50-year-old distressed and angry mother whose 22-year-old son was admitted to the ICU four hours ago following a severe motor vehicle accident.
Setting	You are standing outside the ICU entrance. The afternoon visiting hours have just ended, and the staff has asked you to leave. You refuse to leave because you are frightened and want to see your son immediately.
Emotional state	You are anxious, angry, fearful, and overwhelmed. You feel that nobody is giving you enough information. You believe that hospital rules are preventing you from being with your son when he needs you.
Initial behavior	At the beginning of the simulation, speak loudly and emotionally. Demand to see your son. Question why the nurse is not allowing you to enter. Express distrust, fear, and frustration. Do not become physically violent, but show strong verbal distress.
Interaction rule	Respond dynamically to the nurse’s messages. If the nurse uses empathy, active listening, calm language, and clear explanations, gradually reduce your anger and become more willing to listen. If the nurse is defensive, dismissive, overly technical, rigidly quotes policy without empathy, or threatens security too early, increase your anger and resistance.
Escalation scale	Start at emotional intensity level 8 out of 10. Decrease by one or two levels when the nurse validates your feelings, uses supportive language, and explains boundaries respectfully. Increase by one or two levels when the nurse ignores your emotions, sounds authoritarian, or uses confrontational language.
Response format	Reply with one short realistic statement at a time, as the mother. Do not provide analysis during the role-play. Do not break character unless the learner writes “End simulation.”
Safety boundaries	Do not provide medical advice, diagnosis, prognosis, or treatment recommendations. Do not invent detailed clinical information about the patient. If asked about medical details, respond as the mother by saying that you do not know and that you are scared. Keep the scenario focused on communication and emotional de-escalation.
End of simulation	When the learner writes “End simulation,” stop role-playing and provide structured feedback on the nurse’s communication. Evaluate empathy, active listening, clarity, boundary setting, avoidance of defensive language, and overall de-escalation.

This prompt is intended for educational simulation only. It should not be used with real patient information or as a substitute for supervised communication training.

Simulation workflow

The simulation workflow consists of a sequential process that can be followed by an educator or an individual learner. The learner first reviews the clinical scenario, learning objectives, and safety instructions. Then, the system prompt is entered into ChatGPT to initialize the simulated family-member persona. Once the simulation begins, the learner responds to the family member as a novice critical care nurse, using short written messages that reflect how they would communicate in a real ICU encounter.

During the role-play, ChatGPT responds as the distressed mother according to the escalation and de-escalation rules defined in the system prompt. The interaction may continue for a predefined number of exchanges, such as six to eight learner responses, or until the learner decides to end the simulation. The learner may terminate the scenario at any point by writing “end simulation.” At that stage, ChatGPT stops the role-play and provides structured feedback on the learner’s communication performance.

**Figure 1:**
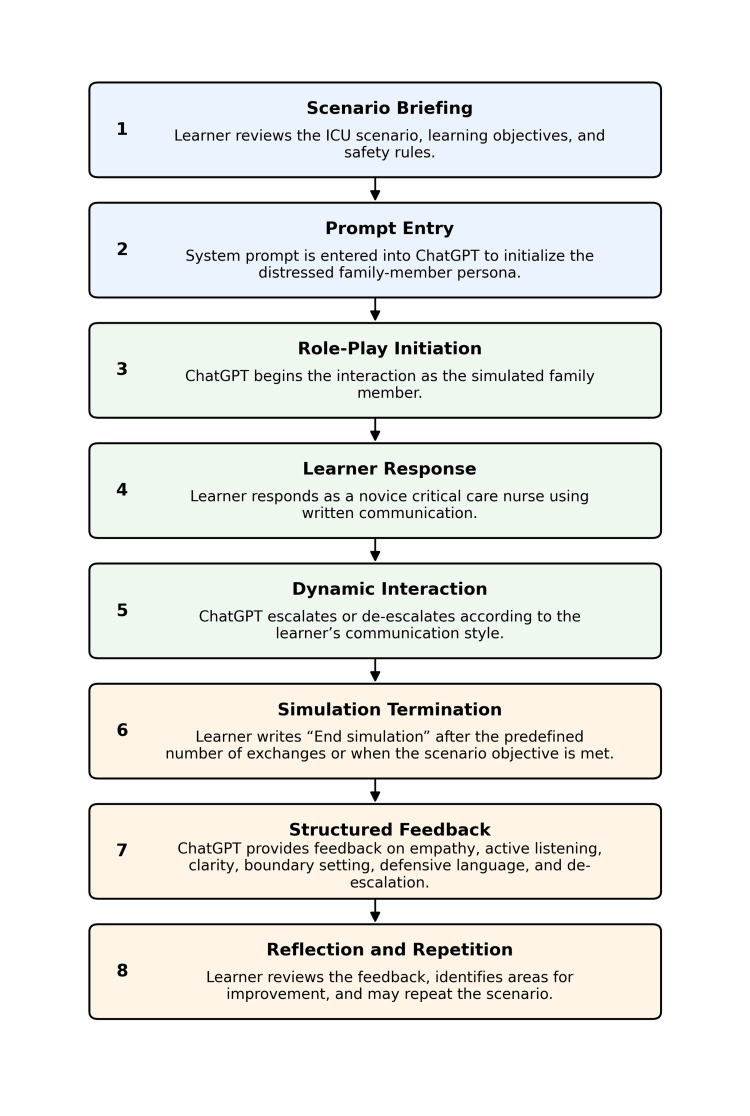
Workflow of the ChatGPT-based ICU family crisis communication simulation protocol The diagram illustrates the eight-step operational process, beginning with the initial scenario briefing and prompt entry, progressing through the dynamic role-play and learner interaction phases, and concluding with automated structured feedback and formative reflection. Figure 1 was created using ChatGPT (OpenAI OpCo, LLC, San Francisco, CA, USA). The content and scientific accuracy of the figure were reviewed and verified by the authors.

The proposed workflow steps from briefing to final reflection are illustrated in Figure 1.

Evaluation and feedback

The evaluation phase begins when the learner ends the role-play by writing “end simulation”. At this point, ChatGPT stops acting as the simulated family member and provides structured feedback on the learner’s communication performance. The feedback is intended to support reflection and skill development rather than to provide a formal pass-or-fail assessment.

The feedback process focuses on six communication domains consisting of empathy, active listening, clarity of communication, professional boundary setting, avoidance of defensive language, and overall de-escalation. For each domain, the model provides a brief comment on the learner’s performance and identifies one practical suggestion for improvement. The learner may repeat the scenario using the same prompt or modify their communication strategy based on the feedback received. The proposed feedback domains and criteria are summarized in Table 3.

**Table 3:** Suggested feedback rubric for learner performance This rubric provides a formative framework for reviewing learner communication after the simulation and should be interpreted alongside educator judgment and learner self-reflection.

Communication domain	Evaluation focus	Example feedback criterion
Empathy	Recognition and validation of emotion	The learner acknowledges the family member’s fear, anger, or uncertainty without dismissing it.
Active listening	Use of reflective and supportive statements	The learner reflects the family member’s concern and demonstrates attention to the emotional message.
Clarity of communication	Simple and understandable explanation	The learner explains the situation and next steps using clear, non-technical language.
Boundary setting	Respectful explanation of limits	The learner explains visiting restrictions or unit rules calmly and without sounding punitive.
Avoidance of defensive language	Non-confrontational response style	The learner avoids blaming, arguing, rigidly quoting policy, or escalating the interaction.
Overall de-escalation	Reduction of emotional intensity	The learner uses communication strategies that help the family member become more willing to listen.

Data privacy and safety considerations

The proposed protocol was designed to avoid the use of real patient information, participant data, or institutional data. The scenario is entirely fictional and should be used only for educational simulation. Learners should be instructed not to enter patient names, medical record numbers, dates of admission, clinical details, staff names, hospital identifiers, or any other information because that information could directly or indirectly identify a patient, a family member, a healthcare professional, or an institution. This is a crucial rule that must be followed by both educator and learner.

Since ChatGPT is a generative AI tool, its responses may vary across sessions even when the same prompt is used. For this reason, the protocol should be used as a formative learning exercise rather than as a formal assessment tool. Educator oversight remains important, particularly when learners receive feedback on sensitive communication issues [8,9]. The feedback generated by ChatGPT should be interpreted as supportive guidance and not as a substitute for faculty judgment, clinical supervision, or institutional communication-training standards [14,15].

The protocol also includes safety boundaries within the system prompt. ChatGPT is instructed not to provide medical advice, diagnosis, prognosis, or treatment recommendations during role-play. If the simulated family member is asked about clinical details, the model is instructed to respond from the perspective of the distressed mother rather than inventing clinical information. These safeguards are intended to keep the simulation focused on communication and de-escalation rather than clinical decision-making.

For responsible implementation, educators should review the prompt, scenario, and feedback rubric before use. Institutions may also adapt the wording of the scenario to align with local policies, visiting rules, and communication-training expectations. Any adaptation should preserve the central educational purpose of the protocol, which allows novice critical care nurses to practice empathic, clear, and non-defensive communication with distressed family members in a controlled, privacy-preserving environment.

## Discussion

The proposed protocol provides a structured way of using ChatGPT as a supplemental simulation tool for communication training in the critical care nursing field. Its main contribution is the development of a simple and reproducible educational structure that allows novice nurses to rehearse difficult conversations with distressed family members before encountering similar situations in daily clinical practice. This is relevant to ICU care because communication, shared decision-making, and family support are central components of family-centered critical care [1-3].

A practical strength of the protocol is that it addresses a common barrier in communication training, which includes the limited availability of repeated, individualized practice opportunities. Educational interventions can support the development of communication skills in nursing students, and simulation-based verbal de-escalation training has been used to prepare healthcare professionals for difficult interpersonal encounters [4,5]. However, traditional approaches may require faculty time, standardized patients, simulation facilities, and scheduled sessions. A ChatGPT-based format cannot replace these methods, but it may offer an accessible way for learners to practice wording, timing, and structure of their responses before supervised simulation or clinical exposure.

Educational implications

From an educational perspective, the protocol may help novice critical care nurses practice non-technical skills that are essential in emotionally charged ICU encounters. These include recognizing distress, validating emotion, using active listening, setting respectful boundaries, and avoiding defensive language. Such skills are difficult to develop through theoretical instruction alone and often require repetition, feedback, and reflection. The proposed simulation gives the learner a protected space to test different communication choices and observe how these choices may influence the direction of the interaction.

Consistent with Bloom’s revised taxonomy, the protocol supports progression from recognizing distress to applying communication strategies and reflecting on performance [16]. The learner is first expected to identify signs of distress, then apply communication strategies such as empathy, active listening, and boundary setting, and finally evaluate their own performance through feedback. This structure supports the use of the protocol as a formative learning activity rather than as a summative assessment of competence.

Recent literature has described the use of ChatGPT and LLM-based systems in simulation scenario development, nursing education, and intensive care nursing education [6-9]. Other studies have explored LLM-based simulated patients and virtual patient systems for communication practice and automated feedback [10,11]. In this context, the present protocol translates their broader capabilities into a specific ICU family communication scenario. Its value lies in making the process explicit: the scenario, system prompt, workflow, feedback rubric, and privacy safeguards are all described in a reproducible format.

Challenges and limitations

Several limitations should be considered. First, the simulation is text-based and cannot reproduce important non-verbal elements of real communication, such as tone of voice, facial expression, posture, silence, physical distance, or the emotional atmosphere of the ICU entrance. This is an important limitation because communication training in healthcare often requires attention not only to what is said, but also to how it is delivered [4,5]. For this reason, the protocol should not be considered equivalent to standardized patient encounters, supervised role-play, or real bedside communication experience.

Second, as with other LLM-based educational tools, some response variability may occur across sessions; therefore, the protocol uses a standardized scenario, predefined prompt, workflow, and feedback structure to support consistency across repeated simulations [10,11]. Some variation may be useful for practice, because learners may be exposed to different emotional responses. However, variability also limits standardization, particularly if the tool is used in a formal educational setting. Even with these safeguards, educator oversight remains necessary.

Third, LLM-generated feedback may be incomplete, overly general, or occasionally inaccurate. For this reason, feedback generated by ChatGPT should be interpreted as formative guidance rather than as a definitive assessment of communication competence. Prior literature on ChatGPT in nursing and healthcare education has emphasized the need for caution regarding reliability, accuracy, bias, and responsible implementation [8,9]. These concerns are particularly relevant when feedback relates to sensitive communication skills or emotionally charged clinical scenarios [14].

Fourth, privacy and data protection are critical considerations. Although the present protocol uses a fictional scenario and does not require real patient information, learners must be explicitly instructed not to enter identifiable patient, family, staff, or institutional data. This is consistent with broader concerns regarding generative AI use in nursing education and healthcare settings [14,15].

Finally, this technical report does not present outcome data. The protocol has not yet been tested with novice critical care nurses, and no conclusions can be drawn regarding its effectiveness in improving communication skills, reducing learner anxiety, or improving performance in real clinical encounters. The manuscript should therefore be interpreted as a description of a reproducible education protocol rather than as evidence of education effectiveness.

Future directions

Future research should evaluate the feasibility, acceptability, and educational value of the protocol in real training settings. A pilot study with novice critical care nurses could examine outcomes such as learners' satisfaction, perceived communication self-efficacy, confidence in verbal de-escalation, quality of written responses, and educator-rated communication performance.

The protocol could also be adapted to additional ICU communication scenarios, including conflict around visiting restrictions, requests for immediate clinical updates, end-of-life discussions, or communication between relatives and staff. These variations could help learners practice communication across different levels of emotional intensity, health literacy, and family distress.

Future versions may combine text-based LLM simulation with educator-led debriefing, standardized patients, or video-based reflection. This approach is consistent with prior work on LLM-based simulated patients and virtual patient systems for communication practice and feedback [10,11,17]. Broader developments in virtual patient ecosystems and generative AI in nursing education may also support more flexible and scalable simulation designs in the future. Nevertheless, future implementation should preserve the safeguards described in this protocol, including avoidance of real patient data, educator oversight, and cautious interpretation of AI-generated feedback [12,13,15].

## Conclusions

This technical report presents a structured ChatGPT-based simulation protocol for training novice critical care nurses in family crisis communication and verbal de-escalation. The protocol includes a fictional ICU scenario, a predefined system prompt, a stepwise workflow, learning objectives, a feedback rubric and privacy safeguards. Its purpose is to offer a low-cost and repeatable practice environment where learners can rehearse empathic, clear, and non-defensive communication with distressed family members. The protocol should be viewed as a supplemental educational framework rather than a replacement for supervised simulation, standardized patient encounters, clinical mentorship, or real-world communication experience. With appropriate oversight and privacy safeguards, LLM-based simulation may provide a practical additional method for supporting communication skills in critical care settings.
